# Effects of fertilization on crop production and nutrient-supplying capacity under rice-oilseed rape rotation system

**DOI:** 10.1038/s41598-017-01412-0

**Published:** 2017-04-28

**Authors:** Muhammad Yousaf, Jifu Li, Jianwei Lu, Tao Ren, Rihuan Cong, Shah Fahad, Xiaokun Li

**Affiliations:** 1 Key Laboratory of Arable Land Conservation (Middle and Lower Reaches of Yangtze River), Ministry of Agriculture; College of Resources and Environment, Huazhong Agricultural University, Wuhan, 430070 China; 2 College of Plant Science and Technology, Huazhong Agricultural University, Wuhan, Hubei 430070 China

## Abstract

Incredible accomplishments have been achieved in agricultural production in China, but many demanding challenges for ensuring food security and environmental sustainability remain. Field experiments were conducted from 2011–2013 at three different sites, including Honghu, Shayang, and Jingzhou in China, to determine the effects of fertilization on enhancing crop productivity and indigenous nutrient-supplying capacity (INuS) in a rice (*Oryza sativa* L.)-rapeseed (*Brassica napus* L.) rotation. Four mineral fertilizer treatments (NPK, NP, NK and PK) were applied in a randomized complete block design with three replicates. Crop yields were increased by 19–41% (rice) and 61–76% (rapeseed) during the two years of rice-rapeseed rotation under NPK fertilization compared to PK fertilization across the study sites. Yield responses to fertilization were ranked NPK > NP > NK > PK, illustrating that N deficiency was the most limiting condition in a rice-rapeseed rotation, followed by P and K deficiencies. The highest and lowest N, P and K accumulations were observed under NPK and PK fertilization, respectively. The INuS of the soil decreased to a significant extent and affected rice-rapeseed rotation productivity at each site under NP, NK, and PK fertilization when compared to NPK. Based on the study results, a balanced nutrient application using NPK fertilization is a key management strategy for enhancing rice-rapeseed productivity and environmental safety.

## Introduction

Agriculture farming, previously dominated by production, currently has diverse objectives. The study of the environmental impacts of crops, the reduced costs of production and the balanced use of fertilization are among the main objectives of modern agriculture^1^. Rice-upland rotations are important agricultural production systems in South Asian countries^1^, covering an estimated 26.7 million hectares^2^. The rice in rotation is followed by next crop called upland crop. This type of rotation has many different sequences, where numerous grain and industrial crops could be rotated with paddy rice. E.g. rice-rapeseed, rice-wheat, rice-potato, rice-Chinese milk vetch etc. In China and other Asian countries, continuous rice planting has had a negative impact on soil properties, such as reduced soil nitrogen supply and organic carbon content^3^. Paddy-rice-upland crop rotations have been recommended and used to improve soil quality and reduce input^3^. In China, these rice-based rotations contribute to 72% of the total cereal production and cover an area of approximately 13 million hectares. Rice (*Oryza sativa* L.) is an important global food crop that ensures food security for many countries. In China, rice is one of the most vital staple food crops, accounting for ~28% of the total grain-sown area and 43% of total grain production^4^. With a constantly growing population, Asian irrigated rice production must increase by 43% over the next 30 years^1^. However, further expansion of the rice planted area is a challenge because a majority of the arable land is already utilized for rice production or has been converted into urban infrastructure^5^. Food security advances must be achieved by constantly improving grain yield per unit area^6^. Oilseed rape (*Brassica napus* L.) is the second most significant source of edible oil globally, with a high nutritional value and a favorable composition of fatty acids for both food and feed^7^. Consequently, its demand is increasing considerably all over the world^8^. China is the leading producer of oilseed rape, containing 23.3% of the cultivated area and contributing 22.2% of global oilseed rape production^1^. Therefore, rice-oilseed rape rotation is of great significance to the economy and food security of China.

The Yangtze River basin in China is a major planting area for the rice-oilseed rape rotation system, contributing 70% of the total rice^9^ and 91% of the total oilseed rape production^10^. Oilseed rape in this area is usually cultivated under the single rice-oilseed rape system or the double rice-oilseed rape system, which limits the crop duration and the soil nutrient supply accessible for increasing yield^11^. Conversely, the yields of rice-upland rotations faced significant decline or yield stagnation with deterioration in soil fertility^12^. Concerns are also increasing about the loss of soil organic matter and the reduced nutrient-supplying capacity of soils under rice-upland rotations, which may be due to the increasing cropping intensity^13^. Farmers have resorted to the use of imbalanced fertilization (i.e., one fertilizer application; for example, in north-central China and the middle and lower reaches of the Yangtze River, only N fertilizer but no P and K fertilizers were applied by farmers, while in northeast China, N and P were applied but not K) or excessive fertilization to maintain yield levels^14–16^. This over-fertilization by farmers, driven by the desire for higher yields, does not always contribute to high yield but it has recently become a common practice for farmers in China nonetheless^15^. Unfortunately, over-fertilization decreases the efficiency of nutrient use^6^ and causes a series of economic and environmental problems^17^. However, balanced mineral fertilizer inputs have played an important role in increasing the rice and oilseed rape yields^18^.

Managing agricultural nutrients to provide a safe food supply and secure the environment remains one of the immense challenges of the 21^st^ century^19^. Crop nutrient uptake and crop yields are the principal factors that determine optimal fertilization practices^20^. Therefore, it is very important to apply fertilizers in an efficient way to minimize loss and to improve the nutrient use efficiency^21^. To the best of our knowledge, previous studies on fertilization responses were rarely conducted on rice and oilseed rape crops individually, and no one has reported fertilization responses in the rice-oilseed rape rotation, particularly in China. In this study, on-farm experiments were conducted at three sites across the Hubei province in central China from May 2011 to May 2013 to study the influence of different mineral fertilizer applications on rice and oilseed rape yield and on the nutrient-supplying capacity of soil during a rice-oilseed rape rotation. These estimates will be helpful for improving fertilizer recommendations and for achieving sustainable production in rice-oilseed rape rotations in central China.

## Results

### Yield of rice and oilseed rape in response to fertilization

The effect of the different fertilization treatments on rice and oilseed rape yield was significant at each study site (Table 1). In 2011–2012 (year one), the rice and oilseed rape yields varied from 5302 to 9048 kg ha^−1^ and 233 to 1687 kg ha^−1^, respectively, while in 2012–2013 (year two), the yields ranged from 4781 to 10746 kg ha^−1^ and 639 to 2600 kg ha^−1^ for rice and oilseed rape, respectively. Depending on the fertilization treatment, overall, the total yield during 2011–2013 (total rotation) varied from 10083 to 19635 kg ha^−1^ and 827 to 4287 kg ha^−1^ for rice and oilseed rape, respectively. Among the sites, the highest rice and oilseed rape yield was observed at the JZ site followed by SY and HH. Compared to PK fertilization, NPK, NK and NP fertilizations significantly increased the rice and oilseed rape yield at each site. At all three sites, the highest rice and oilseed rape yield was observed under NPK application followed by NP and NK, while the lowest yield was observed under PK fertilization. Compared to NPK fertilization, the highest yield reduction was observed under PK followed by NK and NP, at all three sites. These results indicated that fertilization under NPK for rice and oilseed rape was statistically better than the other fertilizer treatments.

**Table 1:** Grain and seed yield (kg ha^−1^) of rice and oilseed rape in different sites of Hubei province affected by with and without NPK fertilization.

Site	Treatment	1^st^ rotation (2011–2012) kg ha^−1^				2^nd^ rotation (2012–2013) kg ha^−1^				Total rotation (2011–2013) kg ha^−1^			
		Rice	% decrease to NPK	oilseed rape	% decrease to NPK	Rice	% decrease to NPK	oilseed rape	% decrease to NPK	Rice	% decrease to NPK	oilseed rape	% decrease to NPK
Honghu	NPK	8603a		1384a		8424a		2060a		17027a		3444a	
	PK	5302b	−38	550d	−60	4781c	−43	793d	−61	10083c	−41	1343d	−61
	NK	8414a	−2	759c	−45	7761b	−8	1172c	−43	16175b	−5	1931c	−44
	NP	8454a	−2	1234b	−11	8009b	−5	1813b	−12	16463b	−3	3047b	−12
Shayang	NPK	9048a		1235a		9286a		2268a		18334a		3503a	
	PK	7784c	−14	233c	−81	5821d	−37	639c	−72	13605d	−26	872c	−75
	NK	8617b	−5	918b	−26	8188c	−12	1893b	−17	16805c	−8	2811b	−20
	NP	8925a	−1	981b	-21	9021b	−3	1970b	−21	17946b	−2	2951b	−16
Jingzhou	NPK	8889a		1687a		10746a		2600a		19635a		4287a	
	PK	8014b	−10	293c	−83	7872c	−27	727b	−72	15886c	−19	1020c	−76
	NK	8768a	−1	850b	−50	10325b	−3	817b	−69	19093b	−3	1667b	−61
	NP	8873a	−0.1	1687a	−0.1	10362b	−4	2537a	−2	19235ab	−2	4224a	−2

Mean values within a column for each season followed by different letters are significantly different at *P* < 0.05 according to LSD.

### Nitrogen, phosphorus and potassium uptake in rice and oilseed rape

Total N, P and K uptake in aboveground plant parts of rice and oilseed rape under different fertilization treatments at each study site are shown in Table 2. Total N uptake was enhanced under NPK fertilization compared with PK treatment. In 2011–2012 (year one), the total N uptake by rice and oilseed rape varied from 82 to 200 kg ha^−1^ (highest uptake at SY site) and 9 to 91 kg ha^−1^ (highest uptake at JZ site), respectively. In 2012–2013 (year two), the total N uptake by aboveground plant parts decreased for rice (72 to 169 kg ha^−1^) and increased for oilseed rape (23 to 108 kg ha^−1^), with the highest N uptake for both recorded at the JZ site. Across the sites, the total N uptake during 2011–2013 (total rotation) was higher for the NPK treatment than for the PK treatment, with the highest uptake at the JZ site for both rice and oilseed rape, followed by the SY and HH sites for rice and the HH and SY sites for oilseed rape.

**Table 2:** Total N, P and K uptake (kg ha^−1^) by aboveground parts of rice and oilseed rape in different sites of Hubei province affected by with and without NPK fertilization.

	Site	Treatment	1^st^ rotation (2011–2012) kg ha^−1^				2^nd^ rotation (2012–2013) kg ha^−1^				Total rotation (2011–2013) kg ha^−1^			
			Rice	% decrease to NPK	oilseed rape	% decrease to NPK	Rice	% decrease to NPK	oilseed rape	% decrease to NPK	Rice	% decrease to NPK	oilseed rape	% decrease to NPK
N	Honghu	NPK	174a		61a		155a		94a		328a		155a	
		PK	82b	−53	20b	−67	72b	−54	34b	−63	154b	−53	55b	−65
	Shayang	NPK	200a		52a		149a		95a		349a		147a	
		PK	144b	−28	9b	−82	82b	−45	23b	−76	226b	−35	32b	−78
	Jingzhou	NPK	183a		91a		169a		108a		352a		199a	
		PK	144b	−21	13b	−85	93b	−45	28b	−74	237b	−33	41b	−79
P	Honghu	NPK	46a		10a		38a		16a		84a		25a	
		NK	40b	−14	6b	−44	35a	−8	9a	−42	75a	−11	15b	−43
	Shayang	NPK	45a		10a		42a		17a		86a		27a	
		NK	40a	−10	7b	−33	39a	−6	13a	−21	79a	−8	20a	−26
	Jingzhou	NPK	43a		13a		45a		21a		88a		34a	
		NK	41a	−4	7b	−51	37a	−19	6b	−72	78a	−11	12b	−64
K	Honghu	NPK	225a		68a		168a		108a		393a		176a	
		NP	199b	−12	58a	−15	157a	−6	85a	−21	356b	−9	143b	−19
	Shayang	NPK	245a		74a		183a		105a		428a		179a	
		NP	245a	−0.2	53b	−28	129b	−30	78b	−26	373b	−13	131b	−27
	Jingzhou	NPK	201a		123a		193a		127a		395a		249a	
		NP	188b	−6	95b	−23	156b	−20	109a	−14	344b	−13	203b	−19

Mean values within a column for each season followed by different letters are significantly different at *P* < 0.05 according to LSD.

Phosphorus uptake in both rice and oilseed rape was greater at all study sites under NPK fertilization compared with NK treatment. Across all sites, P uptake in the total rotation (2011–2013) varied from 75 to 88 kg ha^−1^ and 12 to 34 kg ha^−1^ for rice and oilseed rape, respectively, under NPK fertilization. Compared with NP treatment, potassium uptake in both rice and oilseed rape was enhanced under NPK fertilization in 2011–2012 (year one) and 2012–2013 (year two) at each study site.

Finally, the total K uptake of two consecutive annual rice-oilseed rape rotations (2011–2013) ranged from 344 to 428 kg ha^−1^ for rice and 131 to 249 kg ha^−1^ for oilseed rape under NPK fertilization. Furthermore, the highest N, P and K uptake was observed at the JZ site followed by SY and HH. This illustrated that NPK fertilization treatment was better than the other treatments for improving the N, P and K accumulation of a rice-oilseed rape rotation (Table 3).

**Table 3:** Total N, P, and K uptake (kg ha^−1^) by aboveground part of rice and oilseed rape together in different sites of Hubei province affected by fertilization.

Site	Treatment	Total rotation (2011–2013) kg ha^−1^ (rice +rapeseed)		
		N	P	K
Honghu	PK	208.4d	56.5d	275.8d
	NK	387.6c	89.4c	429.8c
	NP	425.0b	98.6b	499.4b
	NPK	483.1a	109.5a	569.1a
Shayang	PK	258.1d	67.6c	329.2c
	NK	423.1c	99.5b	533.7b
	NP	451.8b	112.6a	504.2b
	NPK	495.4a	113.4a	607.2a
Jingzhou	PK	277.8c	76.3c	357.0d
	NK	431.8b	92.1b	452.7c
	NP	539.4a	115.9a	546.8b
	NPK	550.3a	121.9a	644.0a

Mean values within a column for each season followed by different letters are significantly different at P < 0.05 according to LSD.

### Indigenous nutrient supply for nitrogen, phosphorus and potassium in rice and oilseed rape

**Figure 1:**
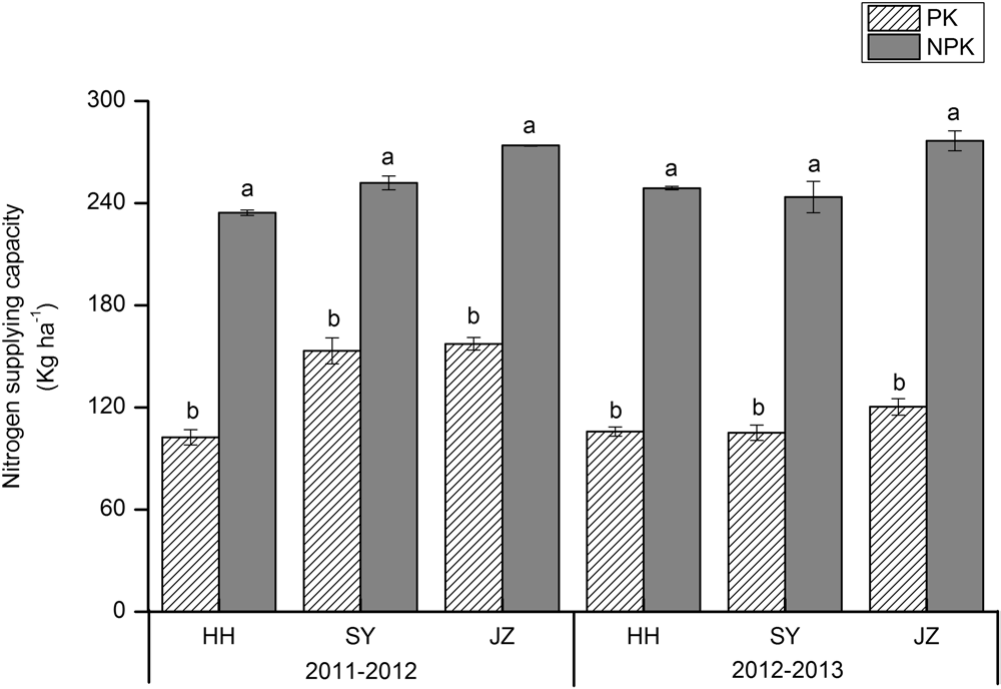
Nitrogen supplying capacity (kg ha^−1^) in 1^st^ (2011–2012) and 2^nd^ (2012–2013) rotation. HH, SY and JZ represent Honghu, Shayang and Jingzhou respectively. Each value represent the standard error (*n* = 3). Within a season, bars with different letters are significantly different at *P* < 0.05 according to LSD.

**Figure 2:**
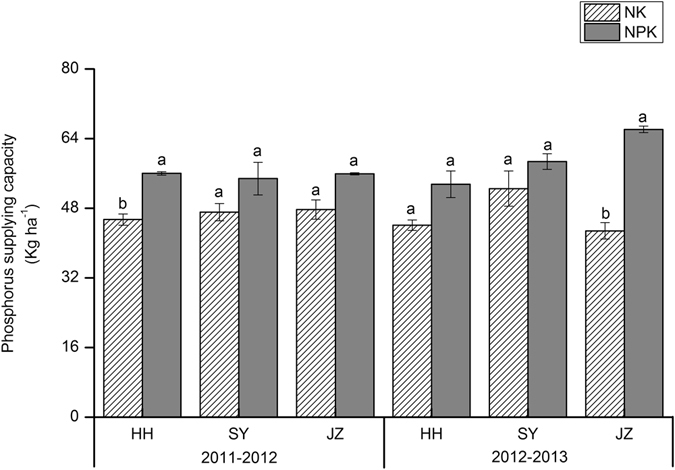
Phosphorus supplying capacity (kg ha^−1^) in 1^st^ (2011–2012) and 2^nd^ (2012–2013) rotation. HH, SY and JZ represent Honghu, Shayang and Jingzhou respectively. Each value represent the standard error (*n* = 3). Within a season, bars with different letters are significantly different at *P* < 0.05 according to LSD.

**Figure 3:**
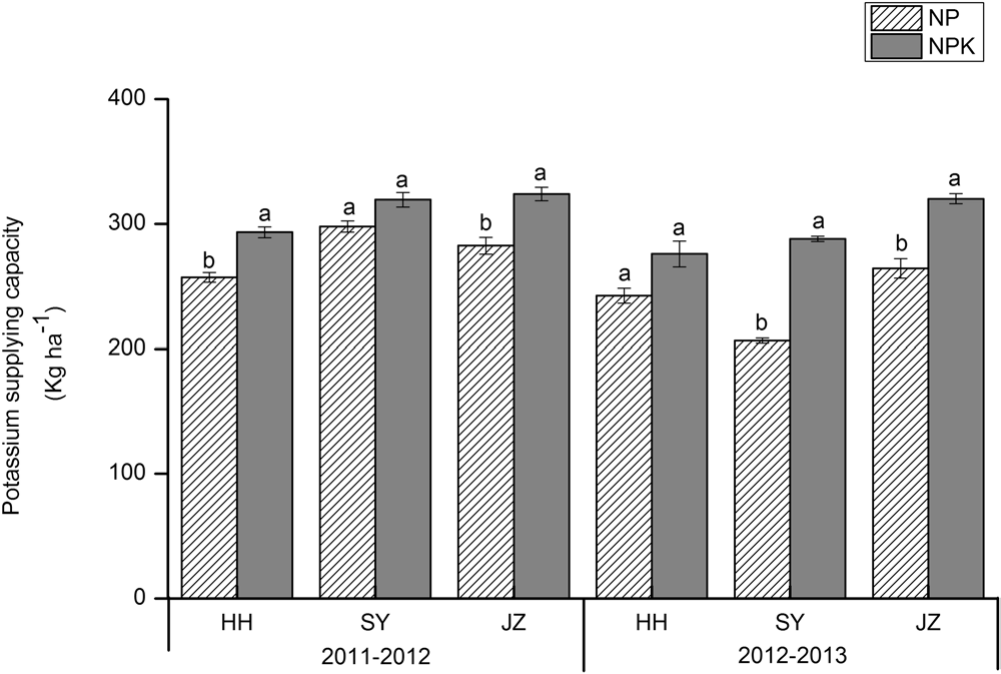
Potassium supplying capacity (kg ha^−1^) in 1^st^ (2011–2012) and 2^nd^ (2012–2013) rotation. HH, SY and JZ represent Honghu, Shayang and Jingzhou respectively. Each value represent the standard error (*n* = 3). Within a season, bars with different letters are significantly different at *P* < 0.05 according to LSD.

In this study, we chose the N, P and K uptake by rice and oilseed rape under no-N, P and K treatment to represent the INS, IPS, and IKS of the soil, respectively. In the first rotation (2011–2012) of rice-oilseed rape across all study sites, INS ranged from 103 to 157 kg N ha^−1^ (Fig. 1), IPS ranged from 45 to 48 kg P ha^−1^ (Fig. 2), and IKS ranged from 257 to 298 kg K ha^−1^ (Fig. 3). In the second rotation (2012–2013), the ranges were 105 to 120 kg N ha^−1^, 43 to 53 kg P ha^−1^, and 206 to 264 kg K ha^−1^ for INS, IPS and IKS, respectively. Compared to NPK fertilization, INS decreased significantly across all sites, by 39–56% in 2011–2012 and 56–57% in 2012–2013 (Fig. 1). IPS decreased by 14–19% and 11–35% in 2011–2012 and 2012–2013, respectively (Fig. 2), while IKS decreased by 7–13% in 2011–2012 and 12–28% in 2012–2013 (Fig. 3), although there were no consistently significant differences between IPS and IKS across all sites. INS and IKS were highest at JZ and lowest at the HH site while IPS was highest at SY and lowest at the HH site.

## Discussion

**Figure 4:**
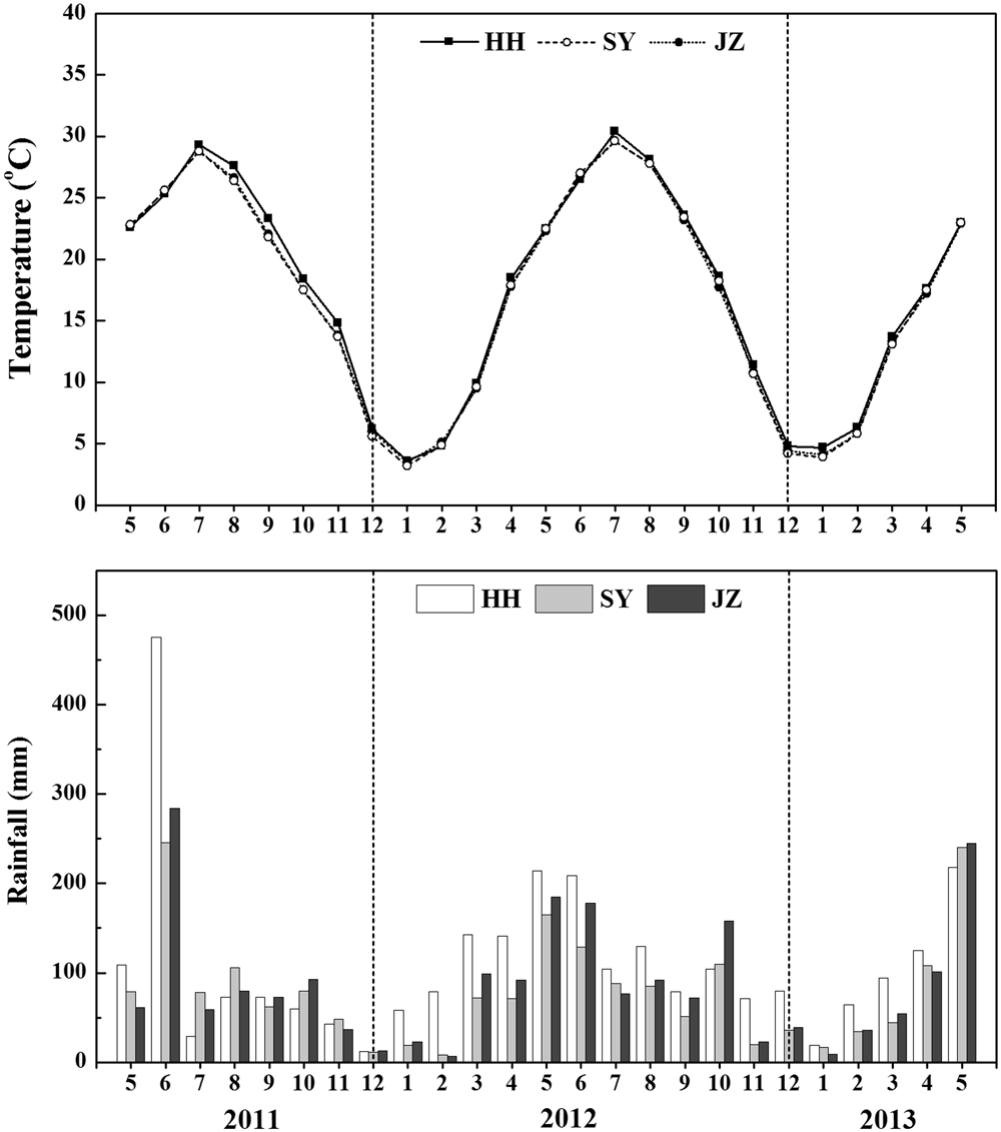
Monthly total rainfall and monthly mean temperature during the crops growth season at the experimental sites in Hubei province of China. HH, SY and JZ represent Honghu, Shayang and Jingzhou respectively.

Sustained crop productivity relies on constant renewal when the supply of nutrients becomes a constraint to plant growth and development. Application of chemical fertilizers is necessary for enhancing crop yields and sustaining soil fertility^12^. However, inappropriate or excessive fertilizer application does not guarantee constantly increasing yields, might result in low nutrient use efficiency, and can cause environmental problems in agro-ecosystems^22, 23^. Compared with PK fertilization, grain and seed yields of both rice and oilseed rape were significantly increased at each site by NPK, NP and NK, which indicates the importance of N for improving crop productivity (Table 1). Among all treatments, NPK fertilization produced the highest yield of both rice-oilseed rape rotations. This high yield was due to the balanced supply of all important nutrients to the plants. Other treatments, such as NP, NK and PK, were lacking at least one major nutrient, i.e., either N, P or K, and thus may induce a specific nutrient deficiency stress and retard overall growth of rice and oilseed rape with a concomitant reduction in yield. Nevertheless, the contributions of chemical fertilizers were diverse. Rice and oilseed rape yields of the 0-N fertilization were significantly lower than those of the 0-P and 0-K fertilizations, showing that N deficiency was the most limiting condition for crop yields^14, 24^, followed by P and K deficiencies. The variations in the yields of rice and oilseed rape at different sites in different years were mainly the consequence of different fertilization treatments associated with soil fertility and the N, P and K uptake ratio by aboveground parts of rice and oilseed rape (Table 2). Furthermore, this discrepancy could also be partly explained by differences in weather conditions and soil physical-chemical properties at different experimental sites (Fig. 4 and Table 4). These results were in agreement with previous studies^25, 26^ that reported that yield components were affected by the fertilizations, and consequently, crop yields were usually greater depending on the soil fertility^27^. A close positive correlation between nutrient uptake and crop yield has also been reported previously^13, 28^. The highest rice and oilseed rape yields were observed at the JZ site, followed by SY and HH, due to their correspondingly higher N, P and K uptakes. In addition to the nutrient uptakes, consideration was also given to their interactions. Many researchers have observed the complicated interactions among N, P and K in crop productivity^29, 30^. In our study, P and K uptake was higher when applied with N, as evidenced by greater P and K accumulation in NPK than in PK treated sites, which clearly indicates the synergistic effect of N on P and K uptake (Table 3). Significantly higher N, P and K uptake was observed under NPK treatment, and the lowest uptake was observed under PK treatment. The remarkable synergistic consequences of N on P^31^ and K^30^ uptake were reported previously. Our results revealed that a balanced NPK treatment was best at improving the yield and nutrient accumulations for rice-oilseed rape rotation. Similar results were documented in a previous study that reported a linear increase in grain yield if nutrients were taken up in balanced amounts, until the yield reached approximately 60 to 70% of the yield potential^32^.

**Table 4:** Locations and soil properties in plow layer (0–20 cm) of three on-farm experimental sites in Hubei province of China.

Site	Coordinate	Soil texture	pH	Organic C	Total N	Olsen-P	NH_4_OA_C_-K
				(g kg^−1^)		(mg kg^−1^)	
Honghu	30°01′N, 113°32′E	Silty clay loam	7.47	24.2	1.93	6.9	96.1
Shayang	31°00′N, 112°24′E	Silt loam	5.88	21.1	1.59	18.9	86.5
Jingzhou	30°20′N, 112°13′E	Silt loam	6.31	26.6	1.97	7.9	98.0

The indigenous nutrient supply of soil can be assessed utilizing different strategies and indicators, including soil properties such as the soil organic C, total N, Olsen-P, NH_4_OA_C_-K, and plant markers such as crop yields and nutrient uptake under a specific nutrient omission treatment^14, 24, 33^. We selected the nutrient uptake by rice and oilseed rape under a specific nutrient omission treatment to represent the INS, IPS and IKS instead of performing a soil test. Many attempts have been made by researchers to predict INuS with a soil test^34, 35^, but soil chemical analysis is not a reliable tool for quantifying nutrient-supplying capacity^36^. The yield decreased significantly when INS decreased (Table 1 and Fig. 1) for rice and oilseed rape at each site during both rotations (2011–2013), indicating that the yield response to N fertilizer is closely associated with INS. A significant correlation was observed between the crop yield and N uptake under no-N treatment for both oilseed rape^14^ and rice^24^. Conversely, the crop yield also decreased by decreasing IPS (Fig. 2) and IKS (Fig. 3), but with an inconsistent significant response, and this reduction was not minimal when compared with yield loss under decreasing INS. However, the IPS and IKS decreased gradually in the second rotation (2012–2013) compared to the first rotation (2011–2012), indicating that P and K fertilizer were still essential for achieving high crop productivity. The variation in INS, IPS and IKS was due to differences in soil chemical properties at the different experimental sites (Table 4). Nutrient dynamics in soil were studied^37^ at different sites, and it was observed that the nutrient fixation, uptake and availability were associated with the content of organic carbon and humic substances in soils. The other components, such as crop, accessibility of other nutrients, nutrient leaching and weather, may also be responsible for the differences in indigenous nutrient supplies. These results demonstrated the impact of INS, IPS and IKS on crop yields, and should therefore be considered during N, P and K fertilization, even though these parameters are frequently ignored^38^. The results reveal that the indigenous nutrient supply of soils is not sufficient to support the intensive cropping system of rice-oilseed rape rotations in central China.

In summary, balanced fertilizer application is not only essential for producing top quality crops in high yields but also for environmental sustainability. Plots that were treated with the combined NPK application had significantly higher rice and oilseed rape yields than plots with no NPK treatment at each experimental site. The lowest yields were observed in the no-N plots (PK), which indicated that N deficiency was the most limiting condition for rice and oilseed rape production. The results revealed that the addition of P and K fertilizer had a considerably positive effect on crop productivity when they were balanced with N. A similar trend was observed for P and K accumulation by aboveground parts of rice and oilseed rape, indicating that it is possible to enhance P and K accumulation when they are applied in combination with N fertilizer. Central China’s agriculture is exhaustive and high yielding and based on multiple cropping systems. The nutrient-supplying capacity of study soils has a significant influence on crop yield and nutrient accumulation. Hence, balanced fertilization of these soils is requisite for avoiding further deterioration of soil fertility and for increasing the productivity of rice-oilseed rape rotations. This fertilization not only improves the yields of the first crop in the rotation but also gives a significant residual advantage to subsequent crops. Agricultural profitability and improved nutrient use efficiency can be achieved through better plant nutrient management, which includes optimum fertilizer applications. Further studies are required on N, P and K fertilizer distribution for rice-oilseed rape rotations to address the variability of economic income and the risk of environmental pollution.

## Methods

### Description of study sites

The two-year field experiment was conducted from 2011 to 2013 on the rice and winter oilseed rape rotation at three different sites in the Hubei province of China: Honghu (HH), Shayang (SY) and Jingzhou (JZ). The previous land uses of these sites were also rice-rapeseed rotations planted by famers. The water requirement for rice was irrigation, while oilseed rape depended on rainfall. The climate of the study regions is a subtropical type, with a mean temperature ranging from 22.3 to 29.4 °C for the two growing seasons of rice and 3.8 to 23.0 °C during the two growing seasons of oilseed rape. Monthly rainfall varied from 10.8 to 475.3 mm in 2011, 7.4 to 213.8 mm in 2012 and 8.7 to 244.6 mm in 2013. During the over-winter oilseed rape cropping season period from January to February, the temperature was usually low (4 °C or lower) with little precipitation (<120 mm) (Fig. 4). The locations of the experimental sites and the soil properties of the plow layers (0–20 cm) at these sites before the commencement of the on-farm experiments are given in Table 4.

### Experimental design and operation

The experiment was conducted in a randomized complete block design in three replicates with four treatments: chemical N, P and K fertilization (NPK); chemical P and K fertilization (PK) with no N; chemical N and K fertilization (NK) with no P; chemical N and P fertilization (NP) with no K. All fertilization treatments in rice and oilseed rape received N as urea at 180 kg ha^−1^ (N 46.4%). The N was applied in three splits with 50% as a base fertilizer, 25% at the tillering stage and 25% at the panicle initiation stage for the rice seasons^24^. The N supplies for the oilseed rape seasons were applied with 60% just before seeding, 20% in the over-wintering stage, and 20% at the initiation of stem elongation^39^. The P fertilizers were applied entirely as a base application at 60 kg ha^−1^ as calcium superphosphate (P 5.2%)^40^ for both the rice and oilseed rape seasons. In the K-fertilized plots, 90 kg ha^−1^ was applied as potassium chloride (K 52.3%)^40^ with applications of 70% before sowing of rice and oilseed rape and 30% either at the panicle initiation stage for rice seasons or at the initiation of stem elongation for oilseed rape seasons. Boron fertilizer (15 kg ha^−1^) was added as a base application in the form of borax (B 12%) for each treatment of the oilseed rape season (but not for rice) to meet its nutritional requirements for normal growth^41^. The plot size for each treatment was 20 m^2^ (3 m × 6.7 m) for both rice and oilseed rape^1^.

The experimental fields at each site were thrice plowed and leveled to an approximately 20-cm depth with a rotary tiller in a dry condition, and the base fertilizers were incorporated during the final plowing. Straw residues were removed before the construction of the experimental plots and the sowing of each crop. Local varieties, Y-liangyou no. 1 and Hua-youza no. 9, widely cultivated in the experimental region with high yields and extensive adaptability, were used for rice and oilseed rape, respectively. The nurseries were raised near the experimental sites on the seed bed with high fertility soils^42^ and were transplanted to the field after 30 days. All other field operations, such as planting density, irrigation, herbicide application and disease and pest control were performed uniformly by methods described previously^42^. No major incidence of weeds, disease, pest or weather was recorded during the growing seasons of the nursery and fields. Planting densities were uniformly set at 200,000 ha^−1^ and 112,500 ha^−1^ for both growing seasons of rice and oilseed rape, respectively. The seeding, transplanting and harvesting times of rice and oilseed rape at each site are shown in Table 5.

**Table 5:** Timing of each operation for rice and oilseed rape in study regions of Hubei province, China.

Operation	Site	1^st^ rotation (2011–2012)		2^nd^ rotation (2012–2013)	
		Rice	Oilseed rape	Rice	Oilseed rape
Seeding	Honghu	8 May 2011	25 Sept. 2011	9 May 2012	20 Sept. 2012
	Shayang	18 Apr. 2011	25 Aug. 2011	18 Apr. 2012	20 Aug. 2012
	Jingzhou	6 May 2011	5 Sept. 2011	12 May 2012	10 Sept. 2012
Transplanting	Honghu	11 Jun. 2011	25 Oct. 2011	12 June 2012	21 Oct. 2012
	Shayang	21 May 2011	25 Sept. 2011	21 May 2012	21 Sept. 2012
	Jingzhou	28 May 2011	5 Oct. 2011	10 June 2012	9 Oct. 2012
Harvesting	Honghu	20 Sept. 2011	15 May 2012	22 Sept. 2012	13 May 2013
	Shayang	10 Sept. 2011	12 May 2012	12 Sept. 2012	11 May 2013
	Jingzhou	13 Sept. 2011	25 May 2012	20 Sept. 2012	5 May 2013

### Sampling and measurement

Sampling and measurements of the soil and plants were conducted using the same protocols across all study sites. Soil samples were collected at a depth of 0–20 cm at 20 random points when each experimental site was established. A sub-sample of fresh soil was used for the measurement of inorganic N^43^. The remaining soil was air-dried and ground to pass through a 2-mm sieve for the measurements of pH (1:2.5 soil/water ratio), organic C (dichromate oxidation method), total N (Kjeldahl acid-digestion method), Olsen-P values (using a spectrophotometer), NH_4_OA_C_-K values (using a flame photometer), and soil type (the hydrometer method).

To investigate the overall effects of the fertilizer applications, plants were sampled at maturity for both crops to determine plant dry matter (kg ha^−1^) and nutrient uptake (kg ha^−1^)^42^. The plant samples were washed with deionized water and divided into seeds, stems, and pod walls for oilseed rape and grains or straw for rice^1^. Each aboveground fraction was separately chopped and dried to a constant weight at 65 °C, and then the entirely dried and milled plant samples were digested with H_2_SO_4_–H_2_O_2_^44^. Total plant N and P concentrations were determined using an automated continuous flow analyzer (AA3, Bran and Luebbe, Norderstedt, Germany). Total plant K concentrations were determined using a flame photometer (FP640). Nutrient uptake was calculated by multiplying the crop dry matter with the nutrient concentrations in the aboveground parts of rice and oilseed rape^40^. At maturity, rice and oilseed rape were harvested manually from each plot, and yields were adjusted to a moisture content of 14% and 8–12%, respectively.

### Data analysis and calculations

Analysis of variance (ANOVA) was conducted on data separated by year. The data were statistically analyzed using the SPSS 17.0 (IBM) software program. The differences between the treatments were calculated using the least significance difference test (LSD) at a 0.05 probability level. Figures were prepared using Origin 8.0 (Origin Lab) software.

Indigenous nitrogen supply (INS) was measured as total plant N accumulation at maturity in 0-N plots, indigenous phosphorus supply (IPS) was measured as total plant P accumulation at maturity in 0-P plots, and indigenous potassium supply (IKS) was measured as total plant K accumulation at maturity in 0-K plots^45^.
